# Supplementary material for: Assessing the Functional Accessibility, Actionability, and Quality of Patient Education Materials from Canadian Cancer Agencies
Source: Curr Oncol. 2023 Jan 19;30(2):1439–49. doi: 10.3390/curroncol30020110 (PMC9955234; doi:10.3390/curroncol30020110)
Supplement: Supplementary file 1 [file curroncol-30-00110-s001.zip › curroncol-2090277-supplementary.pdf]

Supplementary Information Table of Contents

Supplementary Figure S1: Box Plot of Mean Grade Level of Online Patient Education Materials Found on Each Provincial Cancer Agency Using 8 Numerical Scales

Supplementary Table S1. Difficult Words with Alternative Word Recommendations

Supplementary Figure S2: Fry Readability Graph Assessment of Online Patient Education Materials

Supplementary Figure S3. Raygor Readability Estimate Graph of Online Patient Education Materials

Supplementary Table S3. Difficult Words Analysis Statistics

Supplementary Table S4. Readability Formulas

Supplementary Table S5. JAMA Benchmark Criteria

Supplementary Table S6. DISCERN Instrument Criteria

Supplementary Table S7. PEMAT Criteria

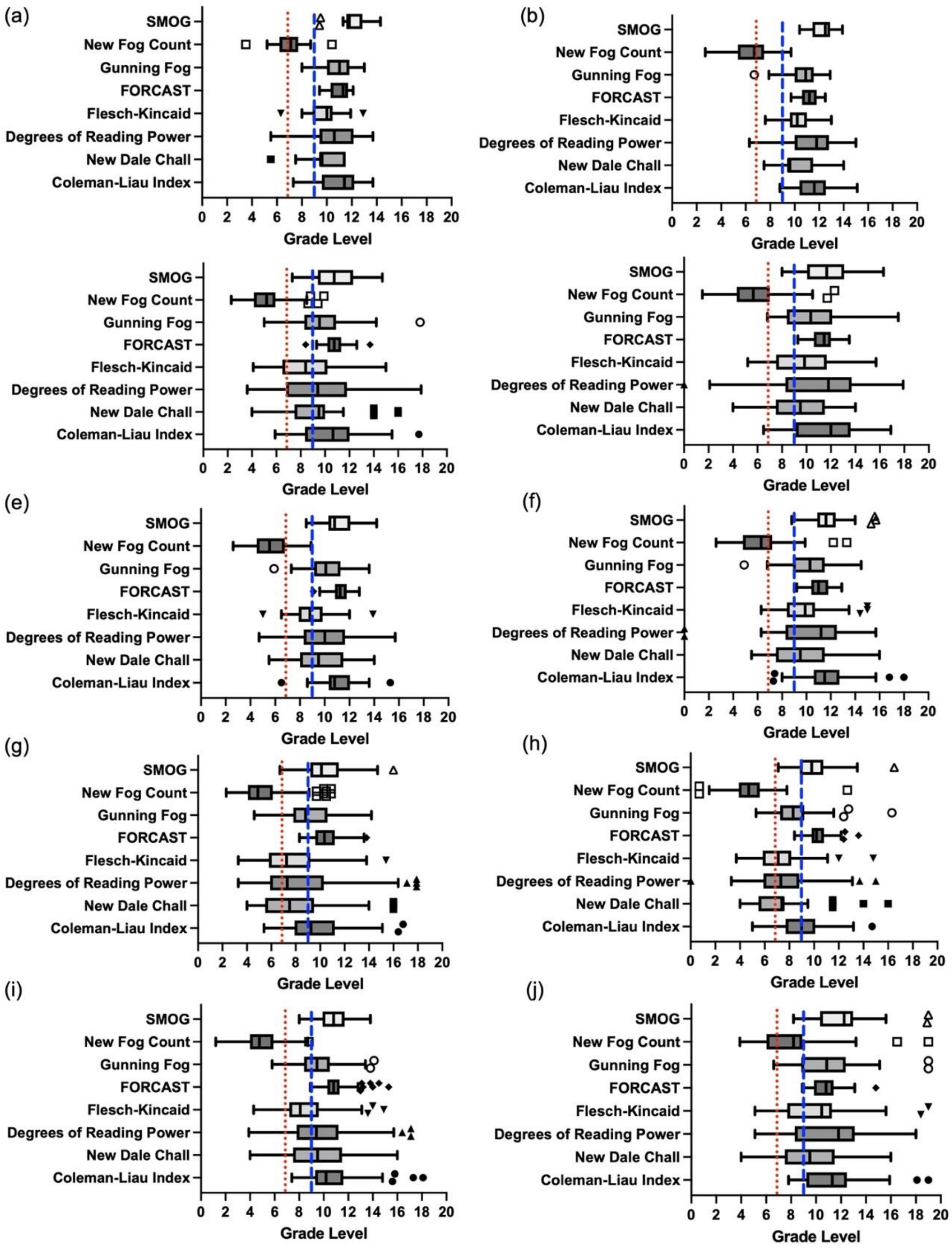

Supplementary Figure S1: Box Plot of Mean Grade Level of Online Patient Education Materials Found on Each Provincial Cancer Agency Using 8 Numerical Scales

Figure S1. The provincial cancer agencies are as follows : (a) New Brunswick, (b) Prince Edward Island, (c) Manitoba, (d) Newfoundland and Labrador, (e) Québec, (f) Saskatchewan, (g) Alberta, (h) Nova Scotia, (i) British Columbia, and (j) Ontario. The blue dashed line in each of the figures represents the average reading level of Canadian adults while the red dotted line represents the recommended reading level of patient education material. Identified outliers are displayed as individual symbols.

Supplementary Table S1. Difficult Words with Alternative Word Recommendations			
**Provincial Cancer Agency**	**Difficult Word***	**Frequency**	**Alternatives*****
New Brunswick (NB)			
	Cervical	109	N/A
	Abnormal (-ly); Abnormality (-ties)	40	Rare; uncommon; unique; not normal; irregular; different
	Nicotine	22	N/A
	Colposcope (-y, -ies)	22	N/A
	Colonoscopy	21	N/A
	Navigate (-or, -tion)	14	Direct; guide
	Vagina (-l)	13	N/A
	Cannabis	9	CBD; THC
	Addiction (-s)	9	N/A
	Colorectal	8	N/A
Prince Edward Island (PEI)			
	Diagnose (-ed, -sis, -ing)	52	Detect; found
	Palliative	45	End of life; symptom support
	Colorectal	31	N/A
	Addiction (-s)	30	N/A
	Cessation	24	Stop
	Navigate (-or, -ion)	24	Direct; guide
	Cervical	23	N/A
	Radiation	23	X-Ray; treatment
	Directive (-s)	23	Order; instruct; wishes
	Practitioner (-s)	21	Doctor
Manitoba (MB)			
	Medication (-s)	395	Drug(-s); treatment(-s); capsule (s); tab (s)
	Radiation	292	X-Ray; treatment
	Palliative	130	End of life; symptom support
	Mammogram	128	N/A
	Chemotherapy	104	Chemo; treatment; drug
	Abnormal (-ly); Abnormality (-ties)	112	Rare; uncommon; unique; not normal; irregular; different
	Constipation (-ed)	106	N/A
	Cervical	100	N/A
	Physician (-s)	100	Doctor
	Diagnose (-ed, -sis, -ing)	94	Detect; found
Newfoundland and Labrador (NL)			
	Radiation	162	X-Ray; treatment
	Palliative	80	End of life; symptom support
	Navigate (-or, -ion)	77	Direct; guide

	Oncology [Oncologist (-s)]	68	Cancer [doctor]
	Diagnose (-ed, -sis, -ing)	53	Detect; find
	Abnormal (-ly); Abnormality (-ties)	49	Rare; uncommon; unique; not normal; irregular; different
	Cervical	48	N/A
	Medication (-s)	38	Chemo; treatment; drug
	Chemotherapy	29	Chemo; treatment; drug
	Terminology	29	Terms; jargon; diction; lingo; phrase
Quebec (QB)			
	Mammogram (-s) [Mammography]	94	Breast exam; breast image
	Asbestos (-is)	71	N/A
	Colorectal	46	N/A
	Cervical	44	N/A
	Vaccination	36	N/A
	Abnormal (-ly); Abnormality (-ties)	23	Rare; uncommon; unique; not normal; irregular; different
	Diagnose (-ed, -sis, -ing)	27	Detect; find
	Contractor	21	Builder; maker; planner
	Genital (-s)	20	Sex organ
	Ministère	20	QB; govt.
Saskatchewan (SK)			
	Mammogram (-s) [Mammography]	55	Breast exam; breast image
	Medication (-s)	53	Drug(-s); treatment(-s); capsule (s); tab (s)
	Radiation	24	X-Ray; treatment
	Oncology [Oncologist (-s)]	24	Cancer [doctor]
	Chemotherapy	22	Chemo; treatment; drug
	Abnormal (-ly); Abnormality (-ties)	21	Rare; uncommon; unique; not normal; irregular; different
	Pharmacy	18	Drugstore
	Antibiotics	16	N/A
	Palliative	15	End of life; symptom support
	Colorectal	15	N/A
Alberta (AB)			
	Radiation/Irradiation	539	X-Ray; treatment
	Medication (-s)	405	Drug(-s); treatment(-s); capsule (s); tab (s)
	Vagina (-l)	229	N/A
	Chemotherapy (-ies)	191	Chemo; treatment; drug

	Oncology [Oncologist (-s)]	184	Cancer [doctor]
	Incision	92	Cut
	Brachytherapy	87	N/A
	Cannabis	80	CBD; THC
	Systemic	79	Whole body; body-wide; system-wide
	Radioactive	78	N/A
Nova Scotia (NS)			
	(Ir-)Radiation	1062	X-Ray; treatment
	Oncology [Oncologist (-s)]	340	Cancer [doctor]
	Medication (-s)	328	Drug(-s); treatment(-s); capsule (s); tab (s)
	Diagnose (-ed, -sis, -ing)	195	Detect; find
	Colorectal	173	N/A
	Caregiver (-s)	160	Carer (s)
	Navigate (-tion) [Navigator (-s)]	130	Direct; guide
	Systemic	110	Whole body; body-wide; system-wide
	Brachytherapy	104	N/A
	Palliative	100	End of life; symptom support
British Columbia (BC)			
	Diagnose (-sis, -ed, -ing)	465	Detect; find
	(Ir-)Radiation	413	X-Ray; treatment
	Lymphoma (-s)	211	Blood cancer; lymph cancer
	Abnormal (-ly); Abnormality (-ties)	179	Rare; uncommon; unique; not normal; irregular; different
	Cervical	143	N/A
	Practitioner (-s)	135	Doctor (-s)
	Systemic	132	Whole body; body-wide; system-wide
	Lymphedema	122	N/A
	Biopsy (-ies)	111	Tissue sample; cell sample
	Chemotherapy	102	Chemo; treatment; drug
Ontario (ON)			
	Radiation	119	X-Ray; treatment
	Diagnose (-ed, -sis, -ing)	119	Detect; find
	Chemotherapy	98	Chemo; treatment; drug
	Palliative	77	End of life; symptom support
	Medication (-s)	74	Drug(-s); treatment(-s); capsule (-s); tab (-s)

Colorectal	52	N/A
Cervical	48	N/A
Constipation (-ed)	42	N/A
Diarrhea	37	N/A
Lymphoma (-s)	34	Blood cancer; lymph cancer

*Inclusion criteria for a "difficult word": 1) Any word with ≥3 syllables that was used at least once in ≥3 patient education material and; 2) was either unlisted on the New Dale Chal list of familiar words and the New General Service List; 3) was not a physical location (e.g. a province, territory, country, or city. **Alternatives selected are those that are considered synonymous while decreasing the individual word(s) syllable and/or character count.

Table S1. Difficult Words with Alternative Word Recommendations: Top 10 most frequent words by provincial cancer agency as described by the difficult word analysis. The frequency of the word in their respective provincial patient education materials (PEMs) and potential alternatives are also depicted.

Supplementary Figure S2. Fry Readability Graph Assessment of Online Patient Education Materials by Provincial Cancer Agency

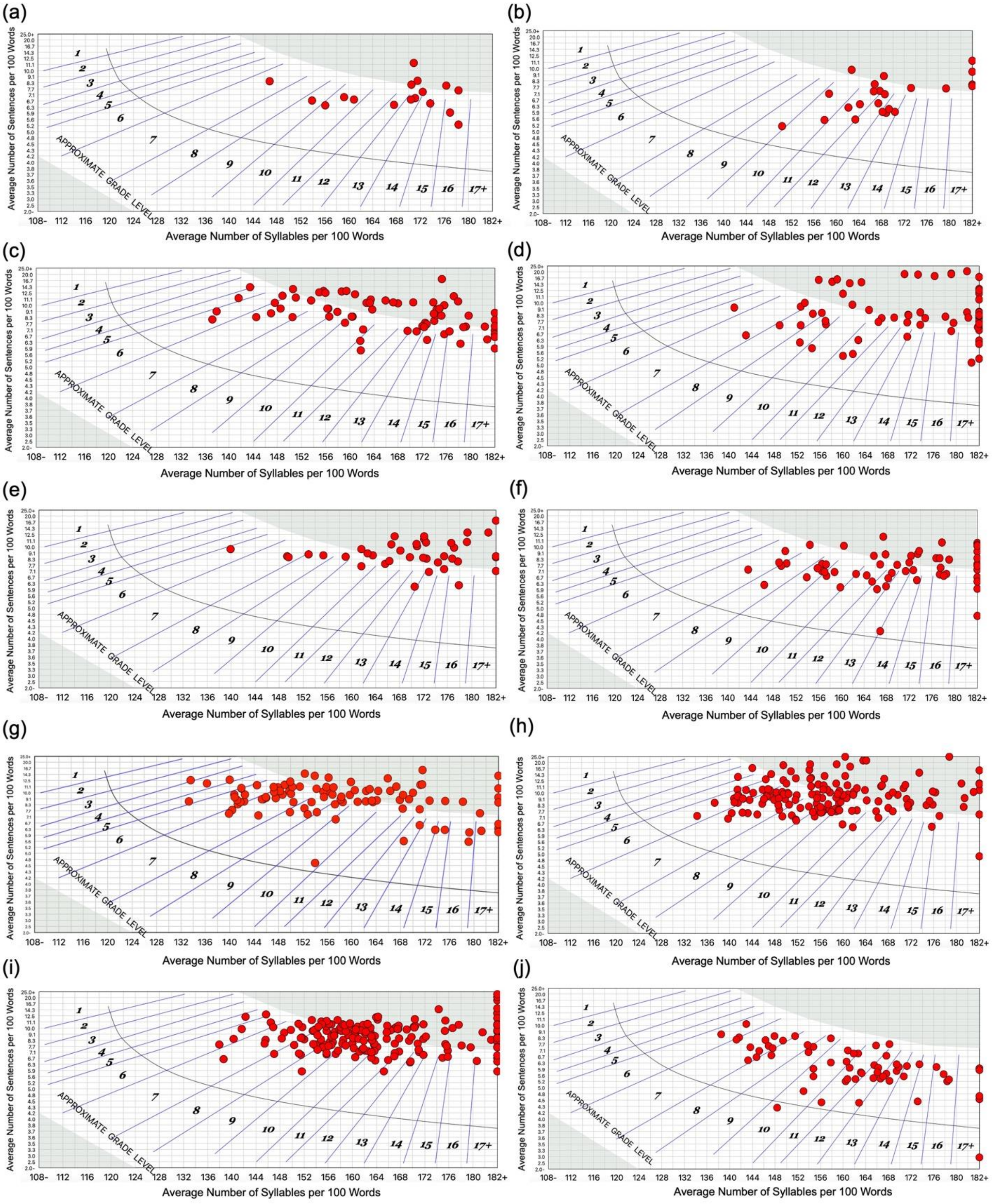

Figure S2. The Fry Readability Graph visually demonstrates the readability of articles by the intersection of the number of syllables per 100 words and the number of sentences per 100 words. Circles indicate reading levels of individual patient education materials (PEMs). The graph was generated using data from PEMs from each of the provincial cancer agencies. The graphs are as follows: (a) New Brunswick, (b) Prince Edward Island, (c) Manitoba, (d) Newfoundland and Labrador, (e) Québec, (f) Saskatchewan, (g) Alberta, (h) Nova Scotia, (i) British Columbia, and (j) Ontario.

Supplementary Figure S3. Raygor Readability Estimate Graph of Online Patient Education Materials by Provincial Cancer Agency

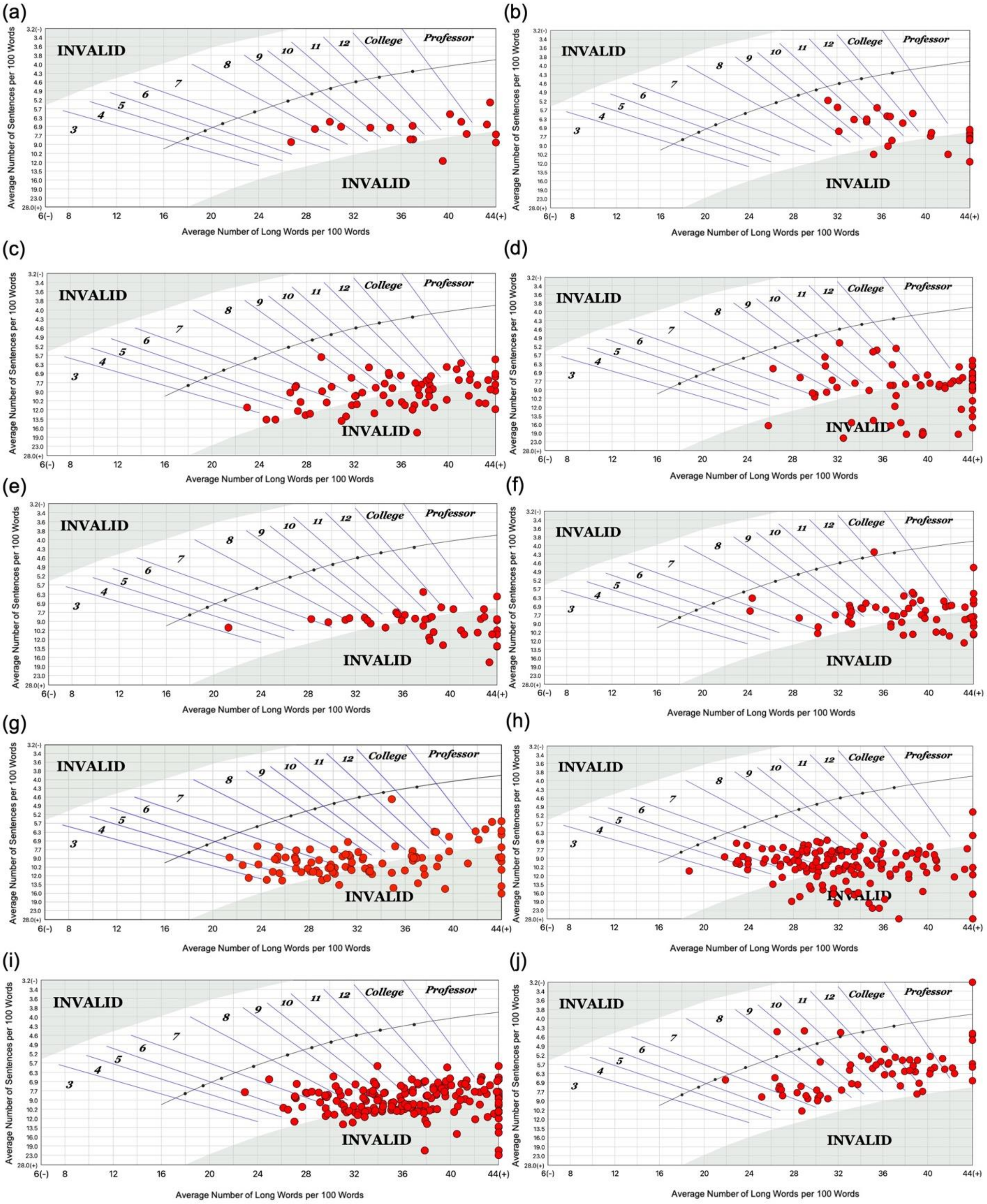

Figure S3. The Raygor Readability Estimate Graph visually demonstrates the readability of the PEMs by the intersection of the number of long words per 100 words

and sentences per 100 words. Numbers within the graph indicate the approximate reading grade level. Circles indicate reading levels of individual patient education materials (PEMs). The graph was generated using data from PEMs from each of the provincial cancer agencies. The graphs are as follows: (a) New Brunswick, (b) Prince Edward Island, (c) Manitoba, (d) Newfoundland and Labrador, (e) Québec, (f) Saskatchewan, (g) Alberta, (h) Nova Scotia, (i) British Columbia, and (j) Ontario.

Supplementary Table S2. Difficult Words Analysis

Provincial Cancer Agency	% 3+ Syllable Words	% 6+ Character words	% Unfamiliar Word*
New Brunswick (NB)			
	16.35 (+/– 3.13)	36.67 (+/– 5.65)	25.15 (+/– 4.49)
Prince Edward Island (PEI)			
	17.33 (+/– 4.21)	39.07 (+/– 5.36)	25.68 (+/– 4.47)
Manitoba (MB)			
	15.84 (+/– 4.91)	35.79 (+/– 5.92)	24.84 (+/– 6.33)
Newfoundland and Labrador (NL)			
	18.61 (+/– 6.21)	39.54 (+/– 6.88)	24.71 (+/– 7.96)
Québec (QB)			
	17.42 (+/– 4.09)	38.05 (+/– 5.85)	26.65 (+/– 5.47)
Saskatchewan (SK)			
	16.75 (+/– 4.19)	38.99 (+/– 6.25)	25.00 (+/– 5.28)
Alberta (AB)			
	14.29 (+/– 7.75)	33.60 (+/– 8.59)	23.15 (+/– 10.91)
Nova Scotia (NS)			
	12.91 (+/– 2.73)	32.17 (+/– 4.72)	20.23 (+/– 5.00)
British Columbia (BC)			
	15.97 (+/– 5.73)	36.81 (+/– 7.65)	26.13 (+/– 6.20)
Ontario (ON)			
	14.52 (+/– 5.38)	35.42 (+/– 6.83)	24.80 (+/– 7.38)

*% Unfamiliar words as determined by the New Dale-Chall criteria. All analyses are reported as the mean and standard deviation (in brackets).

Table S2. Difficult words analysis displaying the mean and standard deviation of the % 3+ syllable words, % 6+ character words, and % unfamiliar words found in the patient education material (PEMs) of each of the provincial cancer agencies.

Supplementary Table S3. Difficult Word Analysis Statistics										
	P Value									
		Pairwise Comparison of Nova Scotia to other cancer agencies' PEMs*								
Difficulty Analysis	Across All PEMs	NB	PEI	MB	NL	QB	SK	AB	BC	ON
3+ Syllables	<0.0001	0.0171	0.0012	0.0011	<0.0001	<0.0001	<0.0001	ns	<0.0001	ns
6+ Characters	<0.0001	ns	<0.0001	0.0034	<0.0001	<0.0001	<0.0001	ns	<0.0001	0.0213
Unfamiliar	<0.0001	0.0165	0.0001	<0.0001	0.0019	<0.0001	<0.0001	0.0265	<0.0001	0.0008

*P values for comparison across the different cancer agencies' PEMs determined using analysis of variance (ANOVA) or equivalent. P values for pairwise comparisons between PEMs was determined using the Tukey's test, or equivalent, for difficulty scores with significant difference ($P < .05$) across the forms on ANOVA. Note: ns indicated non-significance.

Table S3. Analysis of variance (ANOVA) and pairwise comparison of the difficult words analyses [e.g. the % 3+ syllable words, % 6+ character words, and % unfamiliar words found in the patient education material (PEMs) of each of the cancer agencies].

Supplementary table S4. Readability Formulas

Coleman-Liau Index (CLI): 0.0588 x (average number of letters per 100 words) – 0.296 x (average number of sentences per 100 words) –15.8

Degrees of Reading Power (DRP)*: 100 – 100*(0.886593 - 0.083640 x (Number of characters / Number of words) + 0.161911 x (Number of familiar Dale-Chall words / Number of words)^3 – 0.021401 x (Number of words / Number of sentences) + .000577 x (Number of words / Number of sentences)^2 – .000005*(Number of words / Number of sentences)^3)

Flesch-Kincaid Grade Level (FK): 0.39 x (total number of words / total number of sentences) + 11.8 (total number of syllables / total number of words) – 15.59

Ford, Caylor, Sticht (FORCAST): 20-([Number of monosyllabic words per 150 words]/10)

Fry Readability Graph (FRG): a graph plotting the average number of syllables per 100 words on the x-axis and the average number of sentences per 100 words on the y-axis

Gunning Fog Index (GF): 0.4 x ([number of words]/[number of sentences]+(([number of complex words]/[number of words])*100))

New Dale-Chall (NDC): 0.1479 x (number of difficult words / number of words x 100) + 0.0496 x (number of words / number of sentences)

New Fog Count (NFC): (number of easy words + (3 x number of complex words) / (number of senesces) – 3) / 2

Simple Measure of Gobbledygook Index (SMOG): 1.0430 x √(number of polysyllables x [30 / number of sentences]) + 3.1291

Raygor Readability Estimate Graph (RREG): a graph plotting the average number of 6+ characters per 100 words on the x-axis and the average number of sentences per 100 words on the y-axis

*The grade equivalent (GE) of DRP has a corresponding GE according to their DRP score

Table S4. Readability formulas of the 8 numerical and 2 graphical readability tests

Supplementary Table S5. JAMA Benchmark Criteria

Criteria	Description
Authorship	Authors and contributors, their affiliations, and relevant credentials should be provided
Attribution	References and sources for all content should be listed clearly and all relevant copyright information noted
Disclosure	Web site "ownership" should be prominently and fully disclosed as should any sponsorship, advertising, underwriting, commercial funding
Currency	Dates that content was posted and updated should be indicated

Table S5. JAMA benchmark criteria for assessing quality of accountability in education material (PEMs).

Supplementary Table S6. DISCERN Instrument Criteria

1	Are the aims clear?
2	Does it achieve its aims?
3	Is it relevant?
4	Is it clear what sources of information were used to compile the publication?
5	Is it clear when the information used or reported was produced?
6	Is it balanced and unbiased?
7	Does it provide details of additional sources of support and information?
8	Does it refer to areas of uncertainty?
9	Does it describe how each treatment works?
10	Does it describe the benefits of each treatment?
11	Does it describe the risks of each treatment?
12	Does it describe what would happen if no treatment was used?
13	Does it describe how the treatment choices affect overall quality of life?
14	Is it clear that there is more than one treatment choice?
15	Does it provide support for shared decision making?
16	Overall rating

Table S6. DISCERN instrument criteria assessment of the quality and reliability of consumer health information pertaining to treatment choice.

Supplementary Table S7. PEMAT Criteria	
1	The material makes its purpose completely evident.
2	The material does not include information or content that distracts from its purpose.
3	The material uses common, everyday language.
4	Medical terms are used only to familiarize audience with the terms. When used, medical terms are defined.
5	The material uses the active voice.
6	Numbers appearing in the material are clear and easy to understand.
7	The material does not expect the user to perform calculations.
8	The material breaks or "chunks" information into short sections.
9	The material's sections have informative headers.
10	The material presents information in a logical sequence.
11	The material provides a summary.
12	The material uses visual cues (e.g., arrows, boxes, bullets, bold, larger font, highlighting) to draw attention to key points.
15	The material uses visual aids whenever they could make content more easily understood (e.g., illustration of healthy portion size).
16	The material's visual aids reinforce rather than distract from the content.
17	The material's visual aids have clear titles or captions.
18	The material uses illustrations and photographs that are clear and uncluttered.
19	The material uses simple tables with short and clear row and column headings.
20	The material clearly identifies at least one action the user can take.
21	The material addresses the user directly when describing actions
22	The material breaks down any action into manageable, explicit steps
23	The material provides a tangible tool (e.g., menu planners, checklists) whenever it could help the user take action.
24	The material provides simple instructions or examples of how to perform calculations.
25	The material explains how to use the charts, graphs, tables, or diagrams to take actions.
26	The material uses visual aids whenever they could make it easier to act on the instructions.

Table S7. Patient Education Materials Assessment Tool (PEMAT) criteria for assessment of understandability and actionability of patient education material (PEMs).
